# A two-step framework for inferring direct protein-protein interaction network from AP-MS data

**DOI:** 10.1186/s12918-017-0452-y

**Published:** 2017-09-21

**Authors:** Bo Tian, Can Zhao, Feiyang Gu, Zengyou He

**Affiliations:** 1 School of Software, Dalian University of Technology, Tuqiang Road, Dalian, China; 2 Key Laboratory for Ubiquitous Network and Service Software of Liaoning, Tuqiang Road 321, Dalian, 116600 China

**Keywords:** Protein-protein interactions, Affinity purification, Mass spectrometry, Network deconvolution

## Abstract

**Background:**

Affinity purification-mass spectrometry (AP-MS) has been widely used for generating bait-prey data sets so as to identify underlying protein-protein interactions and protein complexes. However, the AP-MS data sets in terms of bait-prey pairs are highly noisy, where candidate pairs contain many false positives. Recently, numerous computational methods have been developed to identify genuine interactions from AP-MS data sets. However, most of these methods aim at removing false positives that contain contaminants, ignoring the distinction between direct interactions and indirect interactions.

**Results:**

In this paper, we present an initialization-and-refinement framework for inferring direct PPI networks from AP-MS data, in which an initial network is first generated with existing scoring methods and then a refined network is constructed by the application of indirect association removal methods. Experimental results on several real AP-MS data sets show that our method is capable of identifying more direct interactions than traditional scoring methods.

**Conclusions:**

The proposed framework is sufficiently general to incorporate any feasible methods in each step so as to have potential for handling different types of AP-MS data in the future applications.

**Electronic supplementary material:**

The online version of this article (doi:10.1186/s12918-017-0452-y) contains supplementary material, which is available to authorized users.

## Background

Proteins play an important role in a variety of biological activities of organism in cells. Knowing the interactions between proteins can facilitate the identification of protein functions and the discovery of new drug targets. Therefore, the accurate inference of protein-protein interaction (PPI) network from experimental data is one of most important and challenging topics in bioinformatics and proteomics.

Affinity purification-mass spectrometry (AP-MS) is a mainstream experimental method for identifying PPIs in a high-throughput manner. In each AP-MS experiment, a tagged protein (bait) is first selectively purified along with its potential interacting partners (preys) from a cell or tissue lysate. Then, MS is used to identify and quantify these affinity purified proteins. Such purification experiments are repeated many times with different bait proteins. The set of bait-prey pairs from all purifications, termed the AP-MS data, is used to infer the underlying protein-protein interaction network structure.

Ideally, one bait protein should have a real and direct interaction relationship with each associated prey protein. However, there is a large number of false positive interactions in the AP-MS data, where the prey protein can be a non-specific contaminant. In addition, some prey proteins do not interact with the bait protein directly, which connect to the bait protein via other intermediate proteins. To remove these spurious interactions and improve the quality and reliability of network, many scoring algorithms have been proposed to solve the PPI inference problem from AP-MS data. As summarized in several recent reviews [1–3], these scoring methods can be categorized into two classes according to the underlying assumption on the composition of candidate interactions: spoke and matrix models. Spoke models consider only bait-prey interactions, whereas matrix models additionally incorporate prey-prey pairs into the set of candidate interactions. On the other hand, these methods are developed for handling different types of AP-MS data. For qualitative AP-MS data, scoring methods mainly measure the strength of interactions according to the co-occurrence correlation between proteins. Typical methods in this category include SA [4], PE [5], DC [6], Hart [7] and IDBOS [8]. For quantitative AP-MS data, some methods such as SAINT [9], MiST [10], ComPASS [11], HGSCore [12] infer interactions between proteins by exploring the quantitative information of proteins.

Despite of recent algorithmic advances on inferring PPI networks from AP-MS data, there are still several challenging problems that remain unsolved. In this paper, we focus on one of such questions: Can we accurately infer the direct PPI network from AP-MS data? Note that there are two types of protein interactions: direct (physical, binary) interaction and indirect (co-complex) interaction. Direct interactions are those in which interacting proteins approach closely and bind together in the form of a complex in some biological processes and then perform certain functions [13]. The indirect interaction between two proteins only refers to their functional relationship without the former direct/physical contact. In other words, two proteins with the indirect interaction cooperate to carry out a given task without actually engaging in a physical contact [14]. Mathematically, if the PPI network is represented as a graph, then each edge in the graph corresponds to a direct interaction. Meanwhile, two proteins have an indirect interaction if they are connected in the graph but have no direct edge. However, most existing scoring methods are developed to infer PPI networks whose edges are mixed of direct and indirect interactions. In other words, these methods do not distinguish direct interactions from indirect interactions in the construction of PPI networks. To our knowledge, only a few studies have investigated the problem of constructing a PPI network that is composed of only direct interactions [15–17].

Therefore, it is still highly demanded to develop effective algorithms for inferring direct protein-protein interactions from the AP-MS data. This paper presents a general framework for inferring direct PPIs from AP-MS data. It is composed of two phases: initialization phase and refinement phase. In the initialization phase, we utilize an existing scoring method to generate a PPI network that may contains both direct and indirect interactions. In the refinement phase, we distinguish direct interactions from indirect interactions in the initial PPI networks. Note that this framework is general and very flexible, in which we can use different algorithms in each phase. To demonstrate the feasibility and advantages of our framework, we conduct a series of comprehensive performance studies. In the experiments, we use SA, PE, DC and Hart methods as the scoring methods in the first phase and two indirect interaction removal methods [18, 19] in the second phase. Experimental results show that our method is capable of detecting more direct interactions than traditional scoring methods.

The rest of the paper is organized as follows. “Methods” section describes our PPI network inference framework. “Results and discussion” section presents the experimental results and “Conclusion” section concludes this paper.

## Methods

Here we propose a general initialization-and-refinement framework for inferring the direct PPI network from AP-MS data. The initial idea of such as a two-step procedure has been discussed in our previous work [3], which has been published online since 2014. In addition, some preliminary experimental results have been presented by the corresponding author in the highlight track of ISB 2015. In this paper, we further formalize this idea and conduct extensive empirical studies to demonstrate its feasibility and effectiveness in practice. In the first step, we use the existing interaction scoring methods to generate an initial PPI network that is mixed of direct and indirect interactions. In the second step, we try to remove indirect interactions from the initial network by utilizing the so-called network cleaning methods.

**Fig. 1:**
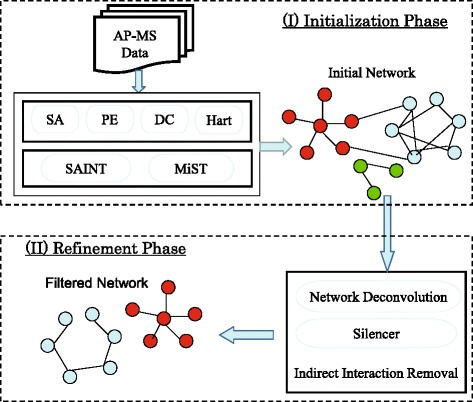
An overview of the two-step framework for direct PPI network inference from AP-MS data. There are two major steps: the initialization step constructs an initial PPI network with the state-of-the-art scoring methods and the refinement step produces a filtered PPI network by removing indirect interactions from the initial network

Figure 1 provides an overview of this framework. In the following, we will elaborate each step in detail.

(I) In the initialization phase, we utilize existing scoring methods to construct an initial PPI network. Indeed, we can use any feasible scoring algorithms in this step. As we have discussed in the introduction, many interaction scoring algorithms have been proposed to infer PPI networks from AP-MS data. These methods are designed to tackle different types of AP-MS data. Therefore, the choice of scoring methods actually depends on the input AP-MS data. For qualitative AP-MS data sets where we only know the co-occurrence of bait-prey pairs, we need to choose methods such as SA [4], PE [5] and DC [6]. For quantitative AP-MS data sets with protein abundance information, we can use those methods such as SAINT [9] and MiST [10].

Despite of the seeming difference among existing methods, the problem of interaction prediction from AP-MS data can be modeled as a complex pairwise correlation mining problem. Here variables correspond to proteins, while samples correspond to purifications. Note that this problem is different from the traditional correlation mining problem with several distinct features: (1) Each variable (i.e.. protein) may take different roles (bait vs. prey). Such information is valuable for effective interaction detection, which has been incorporated in many scoring methods such SA and PE; (2) The data sets are highly noisy, in which many frequently appeared proteins may be containments.

Overall, many existing algorithms are available in the literature that can be utilized in this step. A detailed description and discussion on the advantages and limitations of available methods are beyond the scope of this paper, which could be found in a recent review paper [3].

(II) In the refinement phase, we obtain a filtered PPI network by exploring indirect association cleaning methods on the initial network. Recently, several algorithms have been proposed to recover direct relationships from an observed correlation matrix containing both direct and indirect relationships (e.g. network deconvolution [18], Silencer [19]). Since the initial PPI network generated in the first phase is mixed of direct interactions and indirect interactions, it is feasible to use such indirect association cleaning methods to remove indirect interactions in this phase.

Although these association cleaning methods are developed from quite different starting points, their objectives are the same: inferring the underlying unknown true direct network from the the measured correlation matrix that may be mixed of direct and indirect associations. The basic idea of these methods is summarized as follows.

Suppose a network is represented as an observed pairwise correlation matrix *G*, which is derived from the measurements of the total effect (both direct effect and indirect effect) of each variable on every other variable. If suppose *S* is the true matrix of direct associations, then each entry of correlation measurement in *G* can be obtained by summing up the direct effects mediated through the direct neighbors of the corresponding variable in the true network *S*. Based on this relationship, both the network deconvolution method [18] and the Silencer method [19] provide an approximate closed-form solution for *S* in terms of *G*. Note that actually both approaches are related to the partial correlation [20], which is the correlation between two variables when the effects of other variables are removed. These two methods scale the inverse correlation matrix in different manners.

## Results and discussion

To demonstrate the efficacy and utility of our framework, we conduct a series of tests with several real data sets. The experimental settings, data sets used and performance evaluation results are given in the following sub-sections.

### Experimental settings

In the experiments, we use SA, PE, Hart and DC methods as the scoring methods in the first phase to construct an initial PPI network. All the interaction scores for each algorithm are generated using the ProCope software [21] (https://drupal.bio.ifi.lmu.de/Complexes/ProCope/) with the default parameter settings. To obtain a filtered network in the second phase, we use the network deconvolution (ND) method [18] (http://compbio.mit.edu/nd/code.html) and the Silencer method [19] (https://figshare.com/articles/Nature_Biotechnology_Silencing_Supplementary_Software/1348220) respectively as the indirect interaction removal method, which are executed with their default parameters.

### Data sets and reference sets

We use two public large-scale yeast AP-MS data sets: Gavin [4] and Krogan [22], whose raw experimental data sets were downloaded from http://interactome-cmp.ucsf.edu/. In addition, we also use a larger combined data set, which is generated from the integration of purifications from the above two data sets. The relevant information on these three data sets are summarized in Table 1.

**Table 1:** The relevant statistics on three AP-MS data sets used in the experiments

	Gavin	Krogan	Combined
Number of purifications	2166	4332	6498
Number of distinct baits	1993	2294	2296
Number of distinct preys	2671	5333	5405
Total number of proteins	2761	5364	5444
Number of bait-prey pairs	22165	79738	101903

For each data set, the number of distinct bait proteins, the number of distinct prey proteins, the number of total proteins, and the number of all bait-prey pairs are listed in the table

Although many databases have been constructed for storing PPIs from different species (e.g. [23–25]), there are still no comprehensive gold standard sets for direct protein interactions in the literature. Here we follow Schelhorn et al. [16] to use three reference sets of experimentally validated binary protein interactions for the performance assessment in the experiments. These reference sets are denoted by Y2H, PCA, and BGS, respectively. The first two sets are collections of binary protein interactions experimentally determined from the Y2H technique [26] and the PCA technique [27]. The third reference set is composed of manually curated yeast interactions supported by literature and is taken from an extensive validation of the Y2H method [26]. The Y2H reference set and BGS reference set are downloaded from http://www.sciencemag.org/content/322/5898/104/suppl/DC1 and the PCA reference set is obtained from http://www.sciencemag.org/cgi/content/full/1153878/DC1.

### Performance evaluation results

**Fig. 2:**
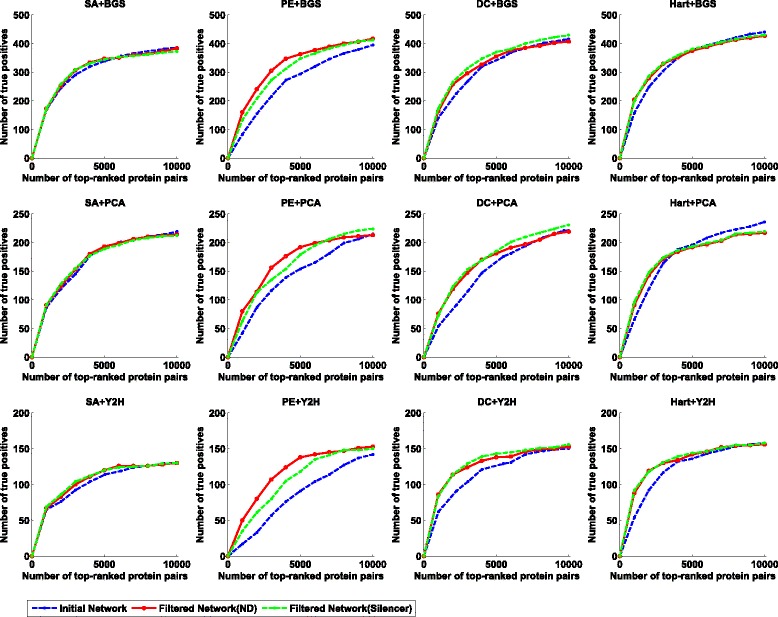
The performance comparison on the Gavin data set when SA, PE, Hart and DC are used as the scoring method in the initial phase and BGS, PCA and Y2H are used as the reference set. To obtain a filtered network, we use both ND and Silencer as the indirect interaction removal method in the second phase. For each reference set and scoring method, we report a set of top-ranked interactions (*x*-axis) to check how many interactions are contained in the the reference set (*y*-axis)

**Fig. 3:**
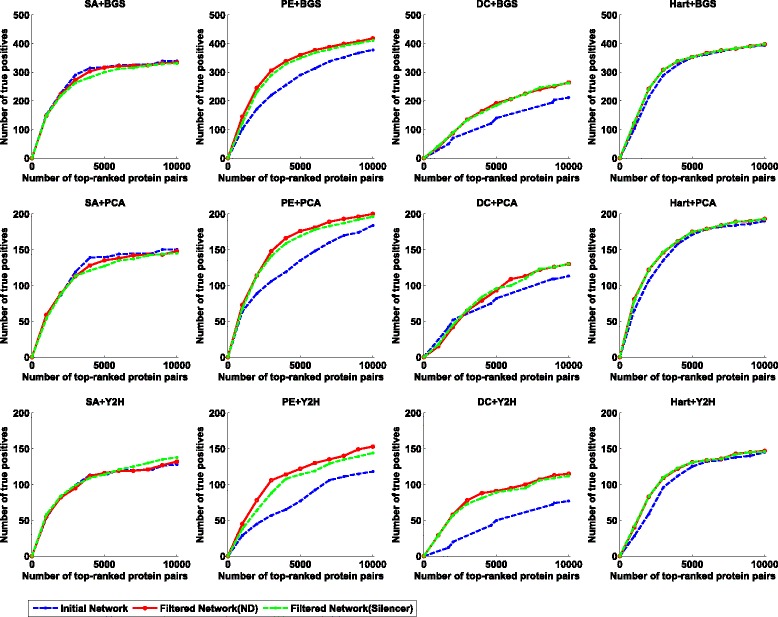
The performance comparison on the Krogan data set when SA, PE, Hart and DC are used as the scoring method in the initial phase and BGS, PCA and Y2H are used as the reference set. To obtain a filtered network, we use both ND and Silencer as the indirect interaction removal method in the second phase. For each reference set and scoring method, we report a set of top-ranked interactions (*x*-axis) to check how many interactions are contained in the the reference set (*y*-axis)

**Fig. 4:**
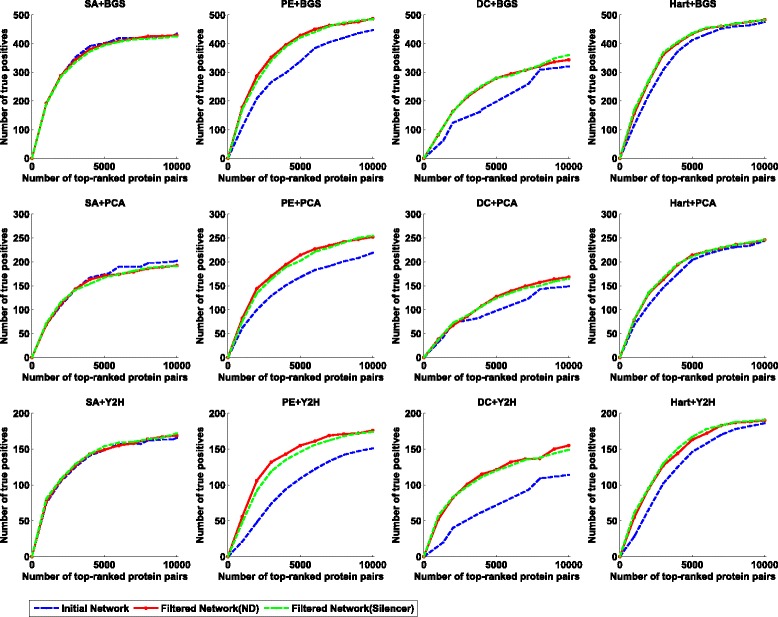
The performance comparison on the Combined data set when SA, PE, Hart and DC are used as the scoring method in the initial phase and BGS, PCA and Y2H are used as the reference set. To obtain a filtered network, we use both ND and Silencer as the indirect interaction removal method in the second phase. For each reference set and scoring method, we report a set of top-ranked interactions (*x*-axis) to check how many interactions are contained in the the reference set (*y*-axis)

To quantify the effectiveness of such an initialization-and-refinement framework, we compare the initial network and filtered network to check if more direct interactions are reported on each data sets. The experimental results on Gavin data, Krogan data, and Combined data are given in Figs. 2, 3, and 4, respectively. As shown in these figures, our two-step method is able to identify more experimentally validated direct interactions than the corresponding initial scoring method in most cases. In order to quantitatively illustrate this fact, we calculate the normalized AUC (area under the curve) value as an overall performance indicator for each method. Here the normalized AUC value is defined as the quotient between the AUC value and *x*_*max*_×*y*_*max*_, where *x*_*max*_ and *y*_*max*_ are the maximal value of *x*-axis and *y*-axis, respectively. The experimental results on three data sets in terms of normalized AUC values are summarized in Tables 2, 3, and 4, respectively. As shown in these tables, the proposed procedure is able to boost the performance of initial networks in terms of normalized AUC values in most cases.

**Table 2:** The performance comparison on the Gavin data set in terms of normalized AUC values

	BGS			PCA			Y2H		
	Initial	Filtered(ND)	Filtered(Silencer)	Initial	Filtered(ND)	Filtered(Silencer)	Initial	Filtered(ND)	Filtered(Silencer)
SA	0.78	0.79 *↑*	0.79 *↑*	0.76	0.76 *↓*	0.76 *↓*	0.77	0.80 *↑*	0.81 *↑*
PE	0.63	0.77 *↑*	0.73 *↑*	0.62	0.74 *↑*	0.71 *↑*	0.54	0.76 *↑*	0.68 *↑*
DC	0.71	0.74 *↑*	0.77 *↑*	0.64	0.70 *↑*	0.72 *↑*	0.73	0.80 *↑*	0.82 *↑*
Hart	0.75	0.77 *↑*	0.77 *↑*	0.73	0.73 *↓*	0.74 *↑*	0.77	0.82 *↑*	0.83 *↑*

In the table, the symbol *↑*highlights the case that the normalized AUC value is increased in the curve derived from the filtered network. If there is no improvement, then *↓* is used instead

**Table 3:** The performance comparison on the Krogan data set in terms of normalized AUC values

	BGS			PCA			Y2H		
	Initial	Filtered(ND)	Filtered(Silencer)	Initial	Filtered(ND)	Filtered(Silencer)	Initial	Filtered(ND)	Filtered(Silencer)
SA	0.82	0.81 *↓*	0.78 *↓*	0.80	0.78 *↓*	0.76 *↓*	0.73	0.73 *↓*	0.76 *↑*
PE	0.62	0.76 *↑*	0.74 *↑*	0.63	0.77 *↑*	0.74 *↑*	0.49	0.72 *↑*	0.66 *↑*
DC	0.47	0.63 *↑*	0.63 *↑*	0.56	0.64 *↑*	0.64 *↑*	0.38	0.71 *↑*	0.68 *↑*
Hart	0.75	0.77 *↑*	0.77 *↑*	0.76	0.79 *↑*	0.79 *↑*	0.71	0.76 *↑*	0.76 *↑*

In the table, the symbol *↑*highlights the case that the normalized AUC value is increased in the curve derived from the filtered network. If there is no improvement, then *↓* is used instead

**Table 4:** The performance comparison on the Combined data set in terms of normalized AUC values

	BGS			PCA			Y2H		
	Initial	Filtered(ND)	Filtered(Silencer)	Initial	Filtered(ND)	Filtered(Silencer)	Initial	Filtered(ND)	Filtered(Silencer)
SA	0.81	0.80 *↓*	0.79 *↓*	0.76	0.73 *↓*	0.73 *↓*	0.76	0.77 *↑*	0.78 *↑*
PE	0.63	0.77 *↑*	0.76 *↑*	0.59	0.74 *↑*	0.72 *↑*	0.55	0.77 *↑*	0.73 *↑*
DC	0.54	0.67 *↑*	0.68 *↑*	0.57	0.67 *↑*	0.65 *↑*	0.44	0.71 *↑*	0.70 *↑*
Hart	0.72	0.77 *↑*	0.78 *↑*	0.70	0.75 *↑*	0.75 *↑*	0.65	0.74 *↑*	0.75 *↑*

In the table, the symbol *↑*highlights the case that the normalized AUC value is increased in the curve derived from the filtered network. If there is no improvement, then *↓* is used instead

To make the discussion easier to follow, we take the experimental results on the Gavin data set as an example for a brief illustration. Table 2 shows the performance comparison between different methods, where total 24 pairs of comparative normalized AUC values are listed. In the table, *↑* highlights the cases that the normalized AUC value is increased in the curve induced from our proposed framework. In contrast, *↓* corresponds to the case without any improvements. Notably, among the 24 pairs of experiments for both ND and Silencer, 21 pairs demonstrate the positive promotion induced by the filtered network, versus only 3 pairs of results with no improvement. Accordingly, our two-step framework can facilitate us to identify more direct interactions compared to the traditional scoring methods.

Similar conclusions can be drawn from the experimental results on the Krogan data set and the Combined data set. More precisely, ND and Silencer can provide at least 19 enhanced cases in both Tables 3 and 4. Moreover, compared to traditional scoring methods in terms of normalized AUC, the worst improvements of the filtered network are 0.02 in Table 3 and 0.01 in Table 4, and the best improvements are 0.33 and 0.27, correspondingly. Meanwhile, in the cases that we cannot achieve performance improvement, the decrease on the performance is almost negligible. This means that it is safe to apply our framework to boost the performance of existing scoring methods for inferring direct PPI networks. In other words, the proposed procedure is able to improve the robustness (i.e., reduce the variance) of final results across a variety of scoring methods.

**Fig. 5:**
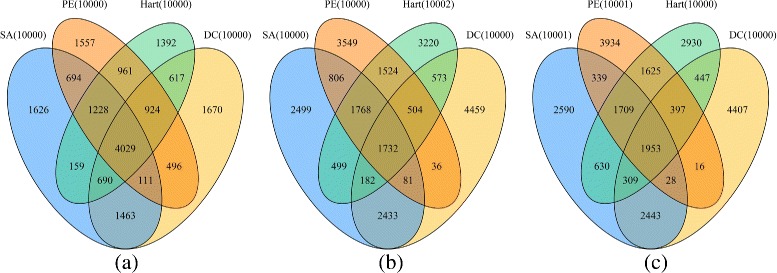
The overlap among the sets of PPIs in the filtered networks derived from the same data set when ND is used in the refinement phase. The results on Gavin, Krogan and Combined data sets are presented in the sub-figure (**a**), (**b**) and (**c**), respectively

**Fig. 6:**
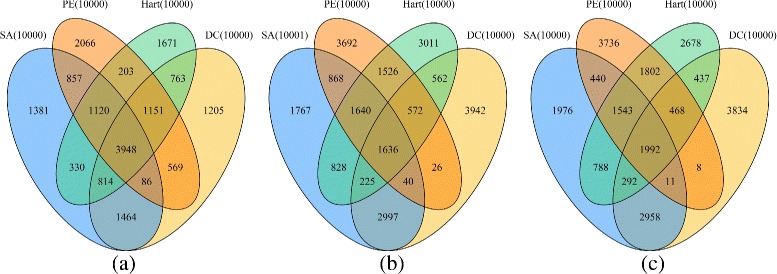
The overlap among the sets of PPIs in the filtered networks derived from the same data set when Silencer is used in the refinement phase. The results on Gavin, Krogan and Combined data sets are presented in the sub-figure (**a**), (**b**) and (**c**), respectively

To check the overlap among the PPIs generated from the same data set by different PPI scoring methods (SA, PE, DC and Hart) after the indirect interaction removal procedure, we plot three Venn diagrams in both Figs. 5 and 6 when ND and Silencer are respectively used as the refinement algorithm. As shown in Fig. 5, we let each scoring method report approximately 10,000 PPIs on each data set. Among these PPIs, the number of PPIs that are reported by all four methods is 4029 (Gaivn), 1732 (Krogan) and 1953 (Combined data), respectively. Moreover, the number of PPIs that are only reported by one method ranges from 1427 to 4622. Therefore, the results obtained by different methods are very diverse. Similar conclusions can be drawn from the Venn diagrams in Fig. 6 as well. This indicates that our proposed framework is applicable to different scenarios.

To illustrate why significant improvements are observed when some scoring methods are used in the first phase, we present the top-10 ranked PPIs and other related details in Additional file 1: Tables S1–S24. When we use ND in the second phase, it is clearly visible that we can always achieve a significant performance improvement over the PE method after the refinement procedure from Tables 2, 3, and 4. This is because many “true PPIs” with low ranks (initial ranks) in Additional file 1: Tables S2, S6, S8 and S10 are re-ranked to be the 10 highest ranked interactions. In contrast, the initial top-10 PPIs and those top-10 ones after refinement are almost the same when the SA method is used in the initialization phase. As a result, the performance improvement is less visible in Tables 2, 3, and 4 when SA is used as the initial scoring method. When Silencer is used in the second phase, similar conclusions can be drawn from Additional file 1: Tables S13–S24 as well.

Overall, our two-step method generally has better performance than the corresponding component scoring methods for each data set. This indicates that the proposed framework is effective in inferring direct PPIs from AP-MS data. Moreover, the refinement step will provide significant performance gain when the results generated from the first step are not good enough. Hence, the two-step framework is of practically considerable value and provides us a new door to conduct the unmixed direct PPI network discovery.

## Conclusion

As AP-MS experiments have generated large amounts of data, it is critical to establish the genuine PPI network from the experimental data. Our two-step framework combines existing PPI scoring methods and network deconvolution techniques, which achieves better performance than the traditional scoring methods on several AP-MS data sets. This framework is sufficiently general to incorporate any feasible methods in each step so as to have potential for handling different types of AP-MS data in the future applications.

In the future work, we will work on optimization models that can infer the direct PPI networks from the AP-MS data in a single procedure. In addition, it is also very critical to develop fast algorithms that can solve the network inference problem in linear time.

## Additional file

Additional file 1 Supplementary Tables. This file provides the supplementary tables (**Tables S1–S24**) that illustrate why significant improvements are observed when some scoring methods are used in the first phase. We present the top-10 ranked PPIs detected from three data sets after the refinement procedure. Meanwhile, we also record the initial ranks of these top-10 ranked PPIs in these tables. Note that we list more than 10 PPIs in some tables such as **Table S3** and **Table S5** because there are top-k ranked PPIs (k >10) that have the same ranking score. (PDF 67 kb)

## Abbreviations

AP-MS: Affinity purification-mass spectrometry
AUC: Area under the curve
BGS: Binary gold standard
COMPASS: Comparative Proteomic Analysis Software Suite
DC: Dice Coefficient
HGScore: Hypergeometric Spectral Counts score
ISB: International Conference on Systems Biology
MiST: Mass Spectrometry Interaction Statistics
ND: Network deconvolution
PCA: Protein fragment complementation assay
PE: Purification Enrichment
PPI: Protein-protein interaction
SA: Socio-Affinity
SAINT: Significance Analysis of the Interactome
Y2H: Yeast two-hybrid
