# Supplementary material for: A two-step framework for inferring direct protein-protein interaction network from AP-MS data
Source: BMC Syst Biol. 2017 Sep 21;11(Suppl 4):82. doi: 10.1186/s12918-017-0452-y (PMC5615237; doi:10.1186/s12918-017-0452-y)
Supplement: Additional file 1 — Supplementary Tables. This file provides the supplementary tables (Tables S1–S24) that illustrate why significant improvements are observed when some scoring methods are used in the first phase. We present the top-10 ranked PPIs detected from three data sets after the refinement procedure. Meanwhile, we also record the initial ranks of these top-10 ranked PPIs in these tables. Note that we list more than 10 PPIs in some tables such as Table S3 and Table S5 because there are top-k ranked PPIs (k >10) that have the same ranking score. (PDF 67 kb) [file 12918_2017_452_MOESM1_ESM.pdf]

Supplementary Document for "A two-step framework for inferring direct protein-protein interaction network from AP-MS data"

Bo Tian, Can Zhao, Feiyang Gu and Zengyou He

Table S1: The top-10 pairs of PPIs detected from the Gavin data set when SA is used as the scoring method in the first phase and ND is used as the refinement algorithm in the second phase. Here "Y" denotes that the PPI is contained in the corresponding reference set.

No	Protein 1	Protein 2	Score	Initial_ rank	BGS	PCA	Y2H
1	YNL104C	YOR108W	1	1			Y
2	YML112W	YKL139W	0.967926	2	Y		
3	YLR226W	YPR161C	0.941676	4	Y		
4	YLR292C	YBR171W	0.939691	3	Y	Y	
5	YPR179C	YNL021W	0.928512	7			
6	YNL126W	YHR172W	0.927059	5	Y		
7	YGR261C	YBR288C	0.925049	13	Y		
8	YNL126W	YLR212C	0.924763	6	Y		Y
9	YNL021W	YDR295C	0.92436	8			
10	YPR049C	YLR423C	0.92429	14			

Table S2: The top-10 pairs of PPIs detected from the Gavin data set when PE is used as the scoring method in the first phase and ND is used as the refinement algorithm in the second phase. Here "Y" denotes that the PPI is contained in the corresponding reference set.

No	Protein 1	Protein 2	Score	Initial_ rank	BGS	PCA	Y2H
1	YGL133W	YOR304W	1	21	Y		
2	YBL045C	YPR191W	0.956684	92	Y		
3	YML049C	YMR288W	0.94869	10			
4	YGL145W	YLR440C	0.9417	42			
5	YOR061W	YGL019W	0.932715	25	Y	Y	
6	YMR061W	YGL044C	0.929286	150			Y
7	YOR061W	YIL035C	0.922944	23	Y		
8	YAR002C-A	YGL200C	0.891438	142			
9	YAL016W	YGL190C	0.886624	156			
10	YER089C	YDR071C	0.876129	407			

Table S3: The top-10 pairs of PPIs detected from the Gavin data set when DC is used as the scoring method in the first phase and ND is used as the refinement algorithm in the second phase. Here "Y" denotes that the PPI is contained in the corresponding reference set.

No	Protein 1	Protein 2	Score	Initial_ rank	BGS	PCA	Y2H
1	YGR093W	YKL149C	1	1			
2	YFR031C	YDR325W	1	1			
3	YJR067C	YNL260C	1	1			
4	YNL072W	YDR279W	1	1			
5	YDR353W	YHR106W	1	1			Y
6	YGR003W	YIL001W	1	1			
7	YJL031C	YPR176C	1	1	Y		Y
8	YJL180C	YIL098C	1	1			
9	YEL041W	YJR049C	1	1			
10	YKL074C	YLR116W	1	1	Y		
11	YML035C	YJL070C	1	1			Y
12	YOR154W	YER140W	1	1			
13	YNR006W	YHL002W	1	1	Y	Y	
14	YLR435W	YLR383W	1	1			
15	YGL153W	YNL214W	1	1		Y	
16	YMR177W	YDR244W	1	1			
17	YPR145W	YGR124W	1	1			
18	YLL041C	YKL148C	1	1	Y		
19	YNL141W	YBR280C	1	1			
20	YOR194C	YKL058W	1	1	Y		Y
21	YNL273W	YMR048W	1	1			Y
22	YCL043C	YHR204W	1	1			

Table S4: The top-10 pairs of PPIs detected from the Gavin data set when Hart is used as the scoring method in the first phase and ND is used as the refinement algorithm in the second phase. Here "Y" denotes that the PPI is contained in the corresponding reference set.

No	Protein 1	Protein 2	Score	Initial_ rank	BGS	PCA	Y2H
1	YDR425W	YJL036W	1	4			
2	YBL045C	YPR191W	0.909387	18	Y		
3	YGL145W	YLR440C	0.846855	12			
4	YAR003W	YLR015W	0.842628	9			
5	YNL258C	YLR440C	0.842481	15			Y
6	YJL208C	YKR079C	0.840706	43			
7	YJL041W	YJL061W	0.835692	31	Y	Y	Y
8	YER089C	YDR071C	0.833819	75			
9	YCR012W	YHR174W	0.833807	44			
10	YAR003W	YPL138C	0.833042	11			

Table S5: The top-10 pairs of PPIs detected from the Krogan data set when SA is used as the scoring method in the first phase and ND is used as the refinement algorithm in the second phase. Here "Y" denotes that the PPI is contained in the corresponding reference set.

No	Protein 1	Protein 2	Score	Initial_ rank	BGS	PCA	Y2H
1	YDR357C	YKL061W	1	1			
2	YOL146W	YDR013W	0.966176	2			
3	YLR417W	YPL002C	0.950656	3	Y		
4	YLR270W	YOR173W	0.942489	4			
5	YJL184W	YKR038C	0.917236	7			
6	YJL072C	YDR013W	0.91489	5			
7	YOL146W	YJL072C	0.913876	6			
8	YOR358W	YBL021C	0.907848	8	Y		
9	YLR423C	YPL166W	0.900358	11			
10	YLR127C	YOR249C	0.895524	10			

Table S6: The top-10 pairs of PPIs detected from the Krogan data set when PE is used as the scoring method in the first phase and ND is used as the refinement algorithm in the second phase. Here "Y" denotes that the PPI is contained in the corresponding reference set.

No	Protein 1	Protein 2	Score	Initial_ rank	BGS	PCA	Y2H
1	YEL056W	YLL022C	1	1			Y
2	YPL001W	YLL022C	0.988951	3			
3	YOR124C	YOR138C	0.959879	66			Y
4	YAR007C	YNL312W	0.927472	73	Y		
5	YPR180W	YDR390C	0.897609	205	Y		
6	YOR123C	YBR279W	0.874476	82			Y
7	YGR086C	YPL004C	0.866927	192			Y
8	YGR103W	YMR049C	0.862425	171			
9	YNL139C	YHR167W	0.861332	129			
10	YLR002C	YOR206W	0.859367	284			

Table S7: The top-10 pairs of PPIs detected from the Krogan data set when DC is used as the scoring method in the first phase and ND is used as the refinement algorithm in the second phase. Here "Y" denotes that the PPI is contained in the corresponding reference set.

No	Protein 1	Protein 2	Score	Initial_ rank	BGS	PCA	Y2H
1	YJR052W	YPL046C	1	1			
2	YDL144C	YFR008W	0.999227	1			
3	YBR300C	YMR222C	0.998757	1			
4	YGL215W	YMR264W	0.990124	1			
5	YDR514C	YJL067W	0.983458	1			
6	YBR292C	YKL051W	0.979918	1			
7	YDL127W	YDL167C	0.975607	1			
8	YOR192C	YGR164W	0.97333	1			
9	YFL003C	YPL232W	0.971116	1			
10	YML107C	YLL067C	0.970216	1			

Table S8: The top-10 pairs of PPIs detected from the Krogan data set when Hart is used as the scoring method in the first phase and ND is used as the refinement algorithm in the second phase. Here "Y" denotes that the PPI is contained in the corresponding reference set.

No	Protein 1	Protein 2	Score	Initial_ rank	BGS	PCA	Y2H
1	YAR009C	YER093C	1	1			
2	YML069W	YGL207W	0.490348	6	Y		
3	YPL001W	YLL022C	0.489197	2			
4	YOR124C	YOR138C	0.484789	19			Y
5	YEL056W	YLL022C	0.476945	3			Y
6	YPL001W	YEL056W	0.472284	4	Y		Y
7	YDR190C	YPL235W	0.469146	8	Y		
8	YAR007C	YNL312W	0.456646	55	Y		
9	YOR123C	YBR279W	0.444414	49			Y
10	YNL201C	YDR075W	0.440328	98			

Table S9: The top-10 pairs of PPIs detected from the Combine data set when SA is used as the scoring method in the first phase and ND is used as the refinement algorithm in the second phase. Here "Y" denotes that the PPI is contained in the corresponding reference set.

No	Protein 1	Protein 2	Score	Initial_ rank	BGS	PCA	Y2H
1	YJR067C	YNL260C	1	1			
2	YDR357C	YKL061W	0.989608	2			
3	YLR292C	YBR171W	0.986076	3	Y	Y	
4	YCL008C	YGR206W	0.95736	4			
5	YOL146W	YDR013W	0.943925	6			
6	YCL008C	YLR119W	0.943142	5			
7	YLR417W	YPL002C	0.942684	7	Y		
8	YOR254C	YLR292C	0.938724	8	Y		
9	YGR206W	YLR119W	0.93269	9			
10	YDR472W	YDR246W	0.909398	10			

Table S10: The top-10 pairs of PPIs detected from the Combine data set when PE is used as the scoring method in the first phase and ND is used as the refinement algorithm in the second phase. Here "Y" denotes that the PPI is contained in the corresponding reference set.

No	Protein 1	Protein 2	Score	Initial_ rank	BGS	PCA	Y2H
1	YOR061W	YIL035C	1	3	Y		
2	YOR061W	YGL019W	0.984179	6	Y	Y	
3	YPL001W	YLL022C	0.966277	70			
4	YAL016W	YGL190C	0.956366	86			
5	YEL056W	YLL022C	0.933005	112			Y
6	YOR124C	YOR138C	0.928986	403			Y
7	YGR103W	YMR049C	0.925426	67			
8	YPL001W	YEL056W	0.916303	146	Y		Y
9	YAR007C	YNL312W	0.914102	256	Y		
10	YBL045C	YPR191W	0.911942	487	Y		

Table S11: The top-10 pairs of PPIs detected from the Combine data set when DC is used as the scoring method in the first phase and ND is used as the refinement algorithm in the second phase. Here "Y" denotes that the PPI is contained in the corresponding reference set.

No	Protein 1	Protein 2	Score	Initial_ rank	BGS	PCA	Y2H
1	YGL153W	YNL214W	1	1		Y	
2	YJR052W	YPL046C	0.997345	1			
3	YGL215W	YMR264W	0.990202	1			
4	YMR157C	YDR106W	0.987425	1			
5	YDR514C	YJL067W	0.983854	1			
6	YBR292C	YKL051W	0.97794	1			
7	YGL109W	YFL020C	0.974288	1			
8	YOR192C	YGR164W	0.972712	1			
9	YML107C	YLL067C	0.967624	1			
10	YGL211W	YDR340W	0.966097	1			

Table S12: The top-10 pairs of PPIs detected from the Combine data set when Hart is used as the scoring method in the first phase and ND is used as the refinement algorithm in the second phase. Here "Y" denotes that the PPI is contained in the corresponding reference set.

No	Protein 1	Protein 2	Score	Initial_ rank	BGS	PCA	Y2H
1	YAR009C	YER093C	1	1			
2	YML085C	YFL037W	0.532992	2			
3	YDR190C	YPL235W	0.525229	3	Y		
4	YPL001W	YLL022C	0.495588	7			
5	YML069W	YGL207W	0.495263	13	Y		
6	YJL130C	YDL055C	0.492382	5			
7	YOR124C	YOR138C	0.490843	20			Y
8	YPL001W	YEL056W	0.483977	9	Y		Y
9	YEL056W	YLL022C	0.481631	11			Y
10	YBR127C	YDL055C	0.476152	4			

Table S13: The top-10 pairs of PPIs detected from the Gavin data set when SA is used as the scoring method in the first phase and Silencer is used as the refinement algorithm in the second phase. Here "Y" denotes that the PPI is contained in the corresponding reference set.

No	Protein 1	Protein 2	Score	Initial_ rank	BGS	PCA	Y2H
1	YNL104C	YOR108W	1	1			Y
2	YML112W	YKL139W	0.954389	2	Y		
3	YLR226W	YPR161C	0.921707	4	Y		
4	YLR292C	YBR171W	0.910223	3	Y	Y	
5	YPR049C	YLR423C	0.909668	14			
6	YGR261C	YBR288C	0.908752	13	Y		
7	YPR179C	YNL021W	0.90578	7			
8	YNL021W	YDR295C	0.900733	8			
9	YAL024C	YHR158C	0.899495	18			Y
10	YDR027C	YDR484W	0.898157	16	Y		

Table S14: The top-10 pairs of PPIs detected from the Gavin data set when PE is used as the scoring method in the first phase and Silencer is used as the refinement algorithm in the second phase. Here "Y" denotes that the PPI is contained in the corresponding reference set.

No	Protein 1	Protein 2	Score	Initial_ rank	BGS	PCA	Y2H
1	YGL133W	YOR304W	0.999739	21	Y		
2	YOR061W	YGL019W	0.948144	25	Y	Y	
3	YOR061W	YIL035C	0.940523	23	Y		
4	YML049C	YMR288W	0.934445	10			
5	YGL145W	YLR440C	0.933506	42			
6	YBL045C	YPR191W	0.90409	92	Y		
7	YKR002W	YNL317W	0.885732	1	Y		
8	YLR115W	YLR277C	0.885074	5	Y		
9	YNL317W	YLR115W	0.883129	4	Y		
10	YKR002W	YLR115W	0.880764	2			

Table S15: The top-10 pairs of PPIs detected from the Gavin data set when DC is used as the scoring method in the first phase and Silencer is used as the refinement algorithm in the second phase. Here "Y" denotes that the PPI is contained in the corresponding reference set.

No	Protein 1	Protein 2	Score	Initial_ rank	BGS	PCA	Y2H
1	YGR093W	YKL149C	1	1			
2	YFR031C	YDR325W	1	1			
3	YJR067C	YNL260C	1	1			
4	YNL072W	YDR279W	1	1			
5	YDR353W	YHR106W	1	1			Y
6	YGR003W	YIL001W	1	1			
7	YJL031C	YPR176C	1	1	Y		Y
8	YJL180C	YIL098C	1	1			
9	YEL041W	YJR049C	1	1			
10	YKL074C	YLR116W	1	1	Y		
11	YML035C	YJL070C	1	1			Y
12	YOR154W	YER140W	1	1			
13	YNR006W	YHL002W	1	1	Y	Y	
14	YLR435W	YLR383W	1	1			
15	YGL153W	YNL214W	1	1		Y	
16	YMR177W	YDR244W	1	1			
17	YPR145W	YGR124W	1	1			
18	YLL041C	YKL148C	1	1	Y		
19	YNL141W	YBR280C	1	1			
20	YOR194C	YKL058W	1	1	Y		Y
21	YNL273W	YMR048W	1	1			Y
22	YCL043C	YHR204W	1	1			

Table S16: The top-10 pairs of PPIs detected from the Gavin data set when Hart is used as the scoring method in the first phase and Silencer is used as the refinement algorithm in the second phase. Here "Y" denotes that the PPI is contained in the corresponding reference set.

No	Protein 1	Protein 2	Score	Initial_ rank	BGS	PCA	Y2H
1	YDR425W	YJL036W	0.999912	4			
2	YBL045C	YPR191W	0.840118	18	Y		
3	YJL208C	YKR079C	0.710801	43			
4	YER089C	YDR071C	0.702438	75			
5	YGL125W	YPL023C	0.668854	122			
6	YJL041W	YJL061W	0.666984	31	Y	Y	Y
7	YCR012W	YHR174W	0.661555	44			
8	YJL061W	YIL115C	0.65859	36		Y	
9	YGL145W	YLR440C	0.656996	12			
10	YNR006W	YHL002W	0.65598	131	Y	Y	

labelS16

Table S17: The top-10 pairs of PPIs detected from the Krogan data set when SA is used as the scoring method in the first phase and Silencer is used as the refinement algorithm in the second phase. Here "Y" denotes that the PPI is contained in the corresponding reference set.

No	Protein 1	Protein 2	Score	Initial_ rank	BGS	PCA	Y2H
1	YDR357C	YKL061W	0.999347	1			
2	YOL146W	YDR013W	0.958951	2			
3	YLR417W	YPL002C	0.95554	3	Y		
4	YLR270W	YOR173W	0.946508	4			
5	YJL184W	YKR038C	0.914184	7			
6	YOR358W	YBL021C	0.90884	8	Y		
7	YJL072C	YDR013W	0.901267	5			
8	YOL146W	YJL072C	0.900986	6			
9	YHR191C	YCL016C	0.89632	17			
10	YLR423C	YPL166W	0.890039	11			

labelS17

Table S18: The top-10 pairs of PPIs detected from the Krogan data set when PE is used as the scoring method in the first phase and Silencer is used as the refinement algorithm in the second phase. Here "Y" denotes that the PPI is contained in the corresponding reference set.

No	Protein 1	Protein 2	Score	Initial_ rank	BGS	PCA	Y2H
1	YEL056W	YLL022C	0.999744	1			Y
2	YPL001W	YLL022C	0.975456	3			
3	YOR124C	YOR138C	0.885041	66			Y
4	YAR007C	YNL312W	0.851099	73	Y		
5	YOR123C	YBR279W	0.796335	82			Y
6	YPR180W	YDR390C	0.778135	205	Y		
7	YPL001W	YEL056W	0.776037	79	Y		Y
8	YNL139C	YHR167W	0.766271	129			
9	YDR190C	YPL235W	0.760152	36	Y		
10	YGR103W	YMR049C	0.75588	171			

Table S19: The top-10 pairs of PPIs detected from the Krogan data set when DC is used as the scoring method in the first phase and Silencer is used as the refinement algorithm in the second phase. Here "Y" denotes that the PPI is contained in the corresponding reference set.

No	Protein 1	Protein 2	Score	Initial_ rank	BGS	PCA	Y2H
1	YJR052W	YPL046C	1	1			
2	YBR300C	YMR222C	0.998195	1			
3	YDL144C	YFR008W	0.997545	1			
4	YGL215W	YMR264W	0.982342	1			
5	YDR514C	YJL067W	0.973941	1			
6	YBR292C	YKL051W	0.970481	1			
7	YDL127W	YDL167C	0.96142	1			
8	YOR192C	YGR164W	0.958524	1			
9	YML107C	YLL067C	0.954585	1			
10	YFL003C	YPL232W	0.95399	1			

Table S20: The top-10 pairs of PPIs detected from the Krogan data set when Hart is used as the scoring method in the first phase and Silencer is used as the refinement algorithm in the second phase. Here “Y” denotes that the PPI is contained in the corresponding reference set.

No	Protein 1	Protein 2	Score	Initial_ rank	BGS	PCA	Y2H
1	YAR009C	YER093C	0.999968	1			
2	YML069W	YGL207W	0.31717	6	Y		
3	YOR124C	YOR138C	0.313126	19			Y
4	YPL001W	YLL022C	0.309356	2			
5	YEL056W	YLL022C	0.289579	3			Y
6	YPL001W	YEL056W	0.280999	4	Y		Y
7	YDR190C	YPL235W	0.276596	8	Y		
8	YAR007C	YNL312W	0.262035	55	Y		
9	YOR123C	YBR279W	0.236983	49			Y
10	YNL201C	YDR075W	0.232943	98			

Table S21: The top-10 pairs of PPIs detected from the Combine data set when SA is used as the scoring method in the first phase and Silencer is used as the refinement algorithm in the second phase. Here “Y” denotes that the PPI is contained in the corresponding reference set.

No	Protein 1	Protein 2	Score	Initial_ rank	BGS	PCA	Y2H
1	YJR067C	YNL260C	0.999997	1			
2	YDR357C	YKL061W	0.983608	2			
3	YLR292C	YBR171W	0.982936	3	Y	Y	
4	YCL008C	YGR206W	0.949029	4			
5	YLR417W	YPL002C	0.941849	7	Y		
6	YOL146W	YDR013W	0.931433	6			
7	YCL008C	YLR119W	0.929377	5			
8	YOR254C	YLR292C	0.924907	8	Y		
9	YGR206W	YLR119W	0.921402	9			
10	YOR358W	YBL021C	0.902363	14	Y		

Table S22: The top-10 pairs of PPIs detected from the Combine data set when PE is used as the scoring method in the first phase and Silencer is used as the refinement algorithm in the second phase. Here "Y" denotes that the PPI is contained in the corresponding reference set.

No	Protein 1	Protein 2	Score	Initial_ rank	BGS	PCA	Y2H
1	YOR061W	YIL035C	0.99789	3	Y		
2	YOR061W	YGL019W	0.972536	6	Y	Y	
3	YPL001W	YLL022C	0.936796	70			
4	YAL016W	YGL190C	0.918933	86			
5	YEL056W	YLL022C	0.892154	112			Y
6	YPL001W	YEL056W	0.864352	146	Y		Y
7	YOR039W	YOR061W	0.849922	51	Y	Y	
8	YAR007C	YNL312W	0.841812	256	Y		
9	YOR124C	YOR138C	0.837755	403			Y
10	YNL139C	YDR138W	0.831323	221			

Table S23: The top-10 pairs of PPIs detected from the Combine data set when DC is used as the scoring method in the first phase and Silencer is used as the refinement algorithm in the second phase. Here "Y" denotes that the PPI is contained in the corresponding reference set.

No	Protein 1	Protein 2	Score	Initial_ rank	BGS	PCA	Y2H
1	YGL153W	YNL214W	1	1		Y	
2	YJR052W	YPL046C	0.995876	1			
3	YGL215W	YMR264W	0.982715	1			
4	YMR157C	YDR106W	0.979842	1			
5	YDR514C	YJL067W	0.973671	1			
6	YBR292C	YKL051W	0.96656	1			
7	YGL109W	YFL020C	0.956681	1			
8	YOR192C	YGR164W	0.956226	1			
9	YML107C	YLL067C	0.949214	1			
10	YGL211W	YDR340W	0.94544	1			

Table S24: The top-10 pairs of PPIs detected from the Combine data set when Hart is used as the scoring method in the first phase and Silencer is used as the refinement algorithm in the second phase. Here "Y" denotes that the PPI is contained in the corresponding reference set.

No	Protein 1	Protein 2	Score	Initial_ rank	BGS	PCA	Y2H
1	YAR009C	YER093C	0.999903	1			
2	YDR190C	YPL235W	0.374234	3	Y		
3	YML085C	YFL037W	0.350732	2			
4	YOR124C	YOR138C	0.332451	20			Y
5	YML069W	YGL207W	0.331923	13	Y		
6	YPL001W	YLL022C	0.320395	7			
7	YPL001W	YEL056W	0.300554	9	Y		Y
8	YEL056W	YLL022C	0.297472	11			Y
9	YJL130C	YDL055C	0.296185	5			
10	YAR007C	YNL312W	0.278156	63	Y		
